# Early and Late Age of Seizure Onset have a Differential Impact on Brain Resting-State Organization in Temporal Lobe Epilepsy

**DOI:** 10.1007/s10548-014-0366-6

**Published:** 2014-06-01

**Authors:** Gaëlle E. Doucet, Ashwini Sharan, Dorian Pustina, Christopher Skidmore, Michael R. Sperling, Joseph I. Tracy

**Affiliations:** 1 Department of Neurology, Thomas Jefferson University, Philadelphia, PA USA; 2 Department of Neurosurgery, Thomas Jefferson University, Philadelphia, PA USA

**Keywords:** Age at seizure onset, Brain organization, Functional connectivity, Temporal lesion, Resting state, fMRI

## Abstract

Temporal lobe epilepsy (TLE) is associated with abnormalities which extend into the entire brain. While the age of seizure onset (SO) has a large impact on brain plasticity, its effect on brain connectivity at rest remains unclear, especially, in interaction with factors such as the presence of mesial temporal sclerosis (MTS). In this context, we investigated whole-brain and regional functional connectivity (FC) organization in 50 TLE patients who underwent a resting-state fMRI scan, in comparison to healthy controls, using graph-theory measures. We first classified TLE patients according to the presence of MTS or not. Then, we categorized the patients based on their age of SO into two subgroups (early or late age of SO). Results revealed whole-brain differences with both reduced functional segregation and increased integration in the patients, regardless of the age of SO and MTS, relative to the controls. At a local level, we revealed that the connectivity of the ictal hippocampus remains the most impaired for an early SO, even in the absence of MTS. Importantly, we showed that the impact of age of SO on whole-brain and regional resting-state FC depends on the presence of MTS. Overall, our results highlight the importance of investigating the effect of age of SO when examining resting-state activity in TLE, as this factor leads different perturbations of network modularity and connectivity at the global and local level, with different implications for regional plasticity and adaptive organization.

**Electronic supplementary material:**

The online version of this article (doi:10.1007/s10548-014-0366-6) contains supplementary material, which is available to authorized users.

## Introduction

Temporal lobe epilepsy (TLE) is the most frequent form of refractory epilepsy, and is commonly associated with mesial temporal sclerosis (MTS). However, there is a growing body of evidence that brain abnormalities in TLE are not limited to the epileptogenic region, but extend into widespread areas of the entire brain. Indeed, this neurological pathology is often referred to as a system disorder with disrupted networks. Consistently, studies have also described these patients as having whole-brain functional and structural connectivity abnormalities (Liao et al. 2010; Zhang et al. 2011). In this context, the age of seizure onset appears to be an important factor potentially influencing brain activity and connectivity, as the disease process interacts with normal developmental changes. Indeed, evidence of altered functional organization depending on the age of seizure onset has been demonstrated through functional MRI (fMRI) and intracarotid amobarbital techniques (Bell et al. 2002; Cousin et al. 2008; Helmstaedter et al. 1997; Helmstaedter 1999).

Furthermore, the effect of an early age of seizure onset has been an essential feature of developmental approaches to understanding the impact of epilepsy and its treatment on cognition and behavior (Helmstaedter et al. 2003). The literature addressing the influence of early seizure onset, while the brain is still highly plastic, relative to later seizure onset is mixed. For instance, when seizures affect a mature brain (e.g. later seizure onset), greater cognitive impairment may be observed (Helmstaedter and Witt 2009). The major explanation for this is that the mature brain is less plastic; allowing late onset seizures to cause irreversible impairment in fully acquired and fully developed cognitive functions. However, the plasticity associated with early development is a double edged sword, as it has also been shown that early seizure onset interferes with brain maturation, resulting in cognitive impairments such as diffuse and mental retardation (Hermann and Seidenberg 2002). Overall, despite the substantial literature demonstrating that age of seizure onset is likely to reorganize brain cognitive networks, there have been no studies yet investigating the impact of this factor on the whole-brain functional connectivity (FC) during the resting-state in TLE. To our knowledge, only one study investigated the relation between inter-hippocampal FC and age of seizure onset in TLE patients, but these authors failed to find a significant association between these variables (Morgan et al. 2011). Thus, to date, using fMRI, the literature investigating the effect of age of seizure onset on brain functional organization and connectivity in TLE is particularly sparse.

While the age of seizure onset appears to have a major influence on brain activity, the presence of MTS is another important factor influencing brain connectivity. While some studies have described more resting-state FC abnormalities in TLE patients with MTS than in non-lesional patients, others have shown that TLE patients without MTS (or other structural lesions) have at least diffuse white matter abnormalities, outside the ictal temporal lobe (Concha et al. 2009; Mueller et al. 2009). Overall, based on distinct patterns of structural change between TLE patients with and without MTS, it has been recently suggested that these two groups possess different epileptogenic networks and, therefore, potentially represent two distinct TLE syndromes (Mueller et al. 2009).

In this project, we sought to fill a gap in this literature, and examine the impact of the age of seizure onset on whole-brain and regional functional connectivity in TLE patients. More specifically, we investigated whole-brain organization in 50 unilateral TLE adult patients who underwent a resting-state fMRI scan. We first distinguished TLE patients according to the presence of a temporal lesion (e.g. MTS) or not. Then, within each TLE group, we categorized patients based on their age of seizure onset (SO) into two subgroups: early/childhood or late/adult age of seizure onset. After investigating global (e.g., whole brain level) changes in our different experimental groups (early/late SO, lesional/non-lesional), we explored the regional organizational properties (in particular in the hippocampus), in order to more precisely localize group differences in brain organization. We hypothesized that our four experimental groups would show distinct patterns of whole-brain organization compared to matched healthy controls, as quantified by topological measures based in graph-theory methodology. We also hypothesized that the presence of a temporal lobe lesion would have a deleterious effect on brain network organization, but, most importantly, this effect would vary with age of seizure onset, as this factor can either constrain or enhance the neuroplastic responses and adaptive resources available to the patients, with early seizure onset potentially conferring an advantage in sustaining more normative organization.

## Materials and Methods

### TLE Patients

A total of 50 refractory unilateral temporal lobe epilepsy patients were recruited from the Thomas Jefferson University Comprehensive Epilepsy Center. Details of the Thomas Jefferson Comprehensive Epilepsy Center algorithm for surgical decision making are described in the study of Sperling et al. (1992). A combination of EEG, MRI, PET, and neuropsychological testing was used to clearly lateralize the side of seizure focus (Sperling et al. 1992). In detail, all patient participants met the following inclusion criteria: unilateral temporal lobe seizure onset through surface video/EEG recordings; concordant PET finding of normal or hypometabolism in the temporal lobe (available for most patients); normal structural MRI or unilateral temporal lesion (mostly MTS; see next section and Table 1). Patients were excluded from the study for any of the following: extratemporal, multifocal or non-localizable epilepsy; bilateral MTS; non-concordant PET with EEG; medical illness with central nervous system impact other than epilepsy; current alcohol or illicit drug abuse; contraindications to MRI; psychiatric diagnosis other than an Axis-I Depression or Anxiety Disorder; or hospitalization for any Axis I disorder listed in the Diagnostic and Statistical Manual of Mental Disorders, IV. Participants provided written informed consent. The study was approved by the Institutional Review Board for Research with Human Subjects at Thomas Jefferson University. Table 1 outlines the demographic, clinical and neuropsychological characteristics of the subjects.

**Table 1:** Clinical information and characteristics of TLE patients and controls

	Lesional TLE patients (mTLE)		Non-lesional TLE patients (nTLE)		Controls
	Early onset (EO)	Late onset (LO)	Early onset (EO)	Late onset (LO)	
N	11	11	14	14	14
Gender	7 F (64 %)	7 F (64 %)	8 F (57 %)	8 F (57 %)	9 F (64 %)
Age (SD, years)	43 (16)	49 (12)	32 (11)	45 (10)	39 (8)
MRI	MTS: 11	MTS: 10	–	–	–
		Cavernous hemangioma: 1			
Mean FSIQ	95	92	92	88	–
Pathology side	6 R (55 %)	6 R (55 %)	8 R (57 %)	8 R (57 %)	–
Illness (epilepsy) duration [year (SD)]	34 (16)	15 (14)	16 (11)	10 (8)	–
Illness onset [years-old (SD)]	9 (6)	33 (10)	17 (5)	35 (8)	–
Min–max	[0–19] year	[20–50] year	[7–24] year	[26-53] year	–
Seizure types	CPS: 3	CPS: 10	CPS: 5	CPS: 5	
	CPS/SPS: 3	CPS/SPS: 0	CPS/SPS: 3	CPS/SPS: 2	
	CPS/rare GS^a^: 5	CPS/rare GS^a^: 1	CPS/rare GS^a^: 1	CPS/rare GS^a^:2	
	CPS w/secGS: 0	CPS w/secGS: 0	CPS w/secGS: 4	CPS w/secGS:4	
		SPS/secGTCS: 0	SPS/secGTCS: 1	SPS/rare CPS: 1	

^a^Less than 10 GS in lifetime. *CPS* complex partial seizures, *SPS* simple partial seizures, *GS* generalized seizures, *secGS* secondarily generalized seizures, *GTCS* generalized tonic/clonic seizures, *FSIQ* full-scale intelligence quotient, *MTS* Mesial temporal sclerosis, *TLE* temporal lobe epilepsy

#### Classification of the TLE Patients

Based on their structural MRI, patients were classified as either non-lesional (nTLE, *N* = 28) or mesial (mTLE, *N* = 22), with the latter mostly referring to the presence of MTS (except one subject having a cavernous hemangioma located in the ictal temporal lobe, see Table 1). Then, based on the age of SO, patients were classified as either “early” or “late” onset (EO or LO, respectively). To maintain balanced onset groups the threshold for classification differed slightly in the nTLE (25 year-old; EO and LO, *n*’s = 14), and mTLE groups (20 year-old; EO and LO, *n*’s = 11) (Table 1).

Of note, the laterality of the ictal focus was not chosen as a third criterion to split the groups, as the sample size would have been too low for meaningful statistical analyses. Instead, we flipped the right-sided TLE patients’ brain along the *y*-*axis*, so that all images were in accordance regarding the site of ictal onset (i.e. on the left side). This step will be described in more detail in the statistical analysis section.

### Healthy Controls

A total of 14 healthy normal controls (NCs, age = 39 ± 8 years-old; 9 females) were also recruited from the Thomas Jefferson University community, matched to the patient participants in age and gender. All controls were free of psychiatric or neurological disorders based on a health screening measure. All controls provided written informed consent.

### Participant MRI Data Acquisition

All participants underwent Magnetic Resonance Imaging on a 3-T X-series Philips Achieva clinical MRI scanner (Amsterdam, the Netherlands) using an 8-channel head coil. A total of 5 min of a resting state condition was collected utilizing Bold Oxygen Level Dependent signal. Anatomical and resting state acquisitions were similar for all participants. A single shot echoplanar gradient echo imaging sequence acquiring T2* signal was used with the following parameters: 120 volumes, 34 axial slices acquired parallel to the AC-PC line, TR = 2.5 s, TE = 35 ms, FOV = 256 mm, 128 × 128 data matrix isotropic voxels, flip angle = 90°, bandwidth = 1.802(±241.1 kHz). The in-plane resolution was 2*2 mm^2^ with a slice thickness of 4 mm. Prior to collection of the T2* images, T1-weighted images (180 slices) were collected using an MPRage sequence (256 × 256 isotropic voxels; TR = 640 ms, TE = 3.2 ms, FOV = 256 mm, flip angle = 8°) in positions identical to the functional scans to provide an anatomical reference. The in-plane resolution for each T1 slice was 1 mm^3^ (axial oblique; angle following AC-PC line). Each EPI imaging series started with three discarded scans to allow for T1 signal stabilization. Subjects lay in a foam pad to comfortably stabilize the head, were instructed to remain still throughout the scan, not fall asleep, and keep their eyes closed during the entire scan.

This study was approved by the Thomas Jefferson Institutional Review Board for Research with Human Subjects, and all participants (patients and controls) provided written informed consent.

### Imaging Processing

Data from the TLE patients and NCs were preprocessed identically using SPM8 (http://www.fil.ion.ucl.ac.uk/spm/software/spm8). Slice timing correction was used to adjust for variable acquisition time over slices in a volume, with the middle slice in every volume used as reference. Next, a six-parameter variance cost function rigid body affine registration was used to realign all images within a session to the first volume. Motion regressors were computed and later used as regressors of no interest. To maximize mutual information, coregistration between the functional scans and MNI305 (Montreal Neurological Institute) template was carried out using six iterations and resampled with a 7th-Degree B-Spline interpolation. Functional images were then normalized into standard space (MNI305) to allow for signal averaging across subjects. We utilized the standard normalization method in SPM8, which minimizes the sum-of-squared differences between the subject’s image and the template (MNI305), while maximizing the prior probability of the transformation. The segmentation of the data into gray matter, white matter (WM), and cerebro-spinal fluid (CSF) classes was then completed. All normalized images were smoothed by convolution with a Gaussian kernel, with a full width at half maximum of 8 mm in all directions. Sources of spurious variance were then removed from the data through linear regression: six parameters obtained by rigid body correction of head motion, the CSF and WM signals. For each individual, the time-courses of both WM and CSF were estimated in the relevant brain tissue classes defined at the segmentation step. Finally, fMRI data were temporally filtered using the REST Toolbox (low cutoff frequency = 0.008 Hz—high cutoff frequency 0.1 Hz) (Song et al. 2011; Cordes et al. 2001).

### ROI Definition

The brain was parcellated into 116 cortical, subcortical and cerebellar regions of interest (ROIs) in MNI space, using a prior anatomical automatic labeling (AAL) atlas (Tzourio-Mazoyer et al. 2002) (see Supplementary Table 1 for the description of the 116 regions). Of note, a ROI may be referred to as a “node”, a term commonly used in graph theory. For each individual, the mean BOLD time-courses from each ROI was computed, by averaging the time series of all voxels within that region. This resulted in a temporal correlation matrix (116*116) for each subject by computing the Pearson correlation coefficients between each pair of ROI time-courses. Each correlation value between two nodes may be referred to as an “edge”. Of note, for the TLE patients having their seizure onset in the right hemisphere, left and right hemispheres’ ROIs were flipped along the y-axis, so that all images were in accordance regarding the site of ictal onset. In other words, after computing the ROI times series and correlation matrix at the individual level, data were collapsed across patients at the group level into results representing the hemisphere ipsilateral and contralateral to the seizure focus (left and right hemisphere for the controls, respectively) (Maccotta et al. 2013).

### Graph Theory Property Computation

For the major whole brain and regional analyses, we chose to investigate three graph theory properties: modularity (M), clustering coefficient (CC), global efficiency (Eglob). Eglob is a measure of functional integration and represents the average inverse shortest path length, thus, functionally it reflects the relative prominence of direct connections between regions without intervening nodes. In other words, each edge will be characterized by an Eglob value. In contrast, both the modularity and the CC are measures of functional segregation. In detail, CC is defined as the fraction of a given node’s neighbors that are neighbors of each other. It is a measure of the density of connections between nearest neighbors of an index node: high clustering coefficients indicate regions that are part of a clique of densely inter-connected neighbors, reflective of better segregation between systems. Each node will be characterized by a CC value. Finally, the modularity property has been defined as the number of edges falling within groups minus the expected number in an equivalent network with edges placed at random (Newman 2006). In other words, it is a whole-brain measure, indicating the degree to which a network may be subdivided into clearly delineated and non-overlapping groups. These measures were calculated employing the Brain Connectivity Toolbox (Rubinov and Sporns 2010).

Similarly to the study of Sequeira et al. (2013), we employed a bootstrapping strategy to evaluate the graph measure differences between the 5 groups. The method used is as follows. Within each group and for each pair of ROIs (e.g., 116*115/2 = 6,670 pairs), individual FC values were submitted to independent sampling with replacement (bootstrapped) across subjects. For each ROI’s pair, across all the participants within a group, bootstrapping was performed 5,000 times, with the mean of each ROI pair computed across the participants, yielding 5,000 connectivity matrices (weighted non-directed, 116*116). Because there is no consensus on the best threshold for such matrices, a set of thresholds was applied, producing a series of binarized matrices. The fixed thresholds ranged from 5 to 35 % of all connections. Graph theory measures were then calculated for each of the 5,000 matrices available at each threshold level. In other words, within each of the matrices for each group only the strongest *n*% of all correlations (n = 5, 10, 15, 20, 25, 35, respectively) were maintained. Depending on the threshold, the resulting graph can be defined either with a high connection density and equivalent to a random graph (i.e., in the case of a low threshold), or with a low connection density, generating disconnected graphs in which some regions might not be linked to any other brain region (i.e., in the case of a high threshold). We limited the threshold to 35 % because increasing this threshold would have included negative FC in the computation of binary matrices for some repetitions.

### Whole-Brain/Global Analyses

The next step involved the calculation of whole network graph metrics for each binary matrix and each group: (1) modularity, (2) average clustering coefficient, and (3) average global efficiency. Then, these whole network measures were submitted to statistical comparisons to evaluate the differences between the 5 experimental groups. Assuming that bootstrapping involved an artificial generation of large samples, and therefore could strongly decrease the mean error of the sample, we decided to apply a very stringent correction for multiple comparisons. We employed a Bonferroni correction based on: the number of parameters evaluated (3), the number of ROI pairs per each matrix (6,670), the number of fixed density of thresholds (6), the number of bootstrapped data (5,000), and the number of tests we conducted (one-way ANOVA, 6) to evaluate the differences between the 5 groups for each parameter. This yielded an effective alpha value of *p* < 1.4 × 10^−11^.

### Region/Local Analyses

Subsequently, we investigated the whole-brain differences between the group at the local level, e.g. at the node level (using CC) and the edge level (using Eglob). However, for more clarity, we only reported the regional analysis on the bootstrapped data binarized at the threshold of 15 % of the connections. We believe 15 % is reasonable, appearing representative of all the thresholds, capturing the common result across the 6 thresholds. However, the CC differences for the other thresholds have been also computed and are displayed in Supplementary Fig. 1 for a general comparison.

**Fig. 1:**
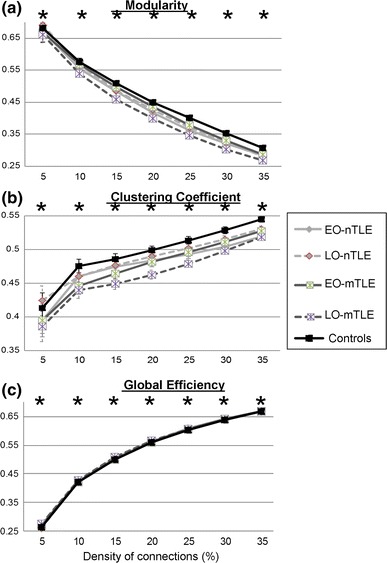
Average whole-brain properties. The graph theory metrics (y-axis) from the bootstrapped data are displayed across fixed density thresholds (x-axis) for binarized matrices. *Black line* control group, *Light gray lines* nTLE groups, *Dark gray lines* mTLE groups, *Bold lines* EO groups, *Dashed lines* LO groups. *Stars* indicate significant differences between groups; result issued from the ANOVAs realized between the 5 groups, for each threshold independently (*p* < 10^−12^). *Error bars* represent the standard deviations computed for the 5,000 repetitions of the bootstrapping analysis. Of note, they are not displayed for the Eglob as they were equal to 0.01 or less, regardless of the groups and the thresholds

**Fig. 2:**
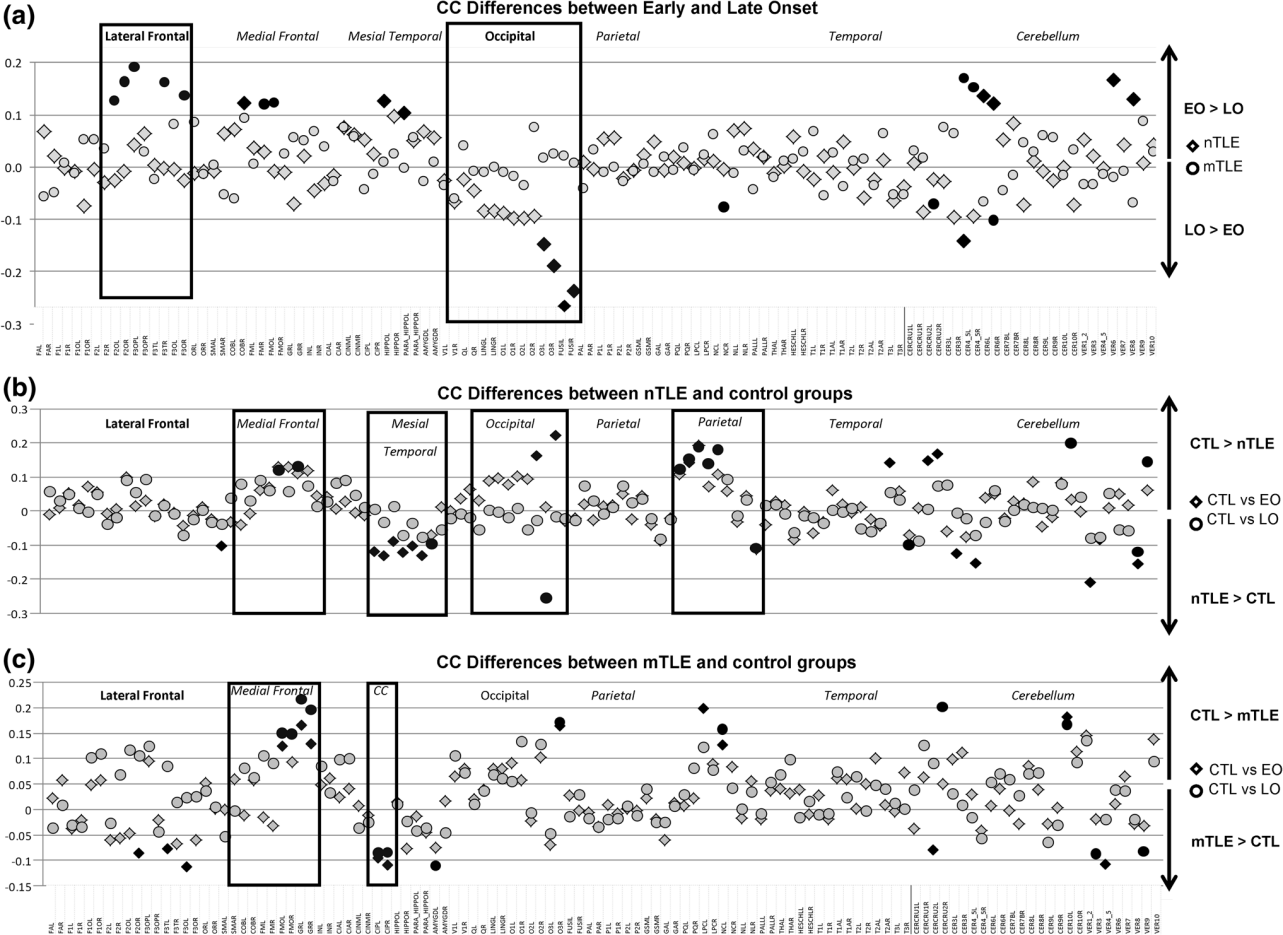
Clustering coefficient differences between the experimental groups, for the 116 ROIs, for the density threshold 15 %. **a** Differences between EO and LO, within each TLE group. **b** Differences between the EO/LO nTLE and the control (CTL) groups, respectively. **c** Differences between the EO/LO mTLE and control groups, respectively. The largest differences are highlighted in *black* (≥m ± 1.5SD). The *abbreviations* of the regions’ name are explained in Supplementary Table 1

**Fig. 3:**
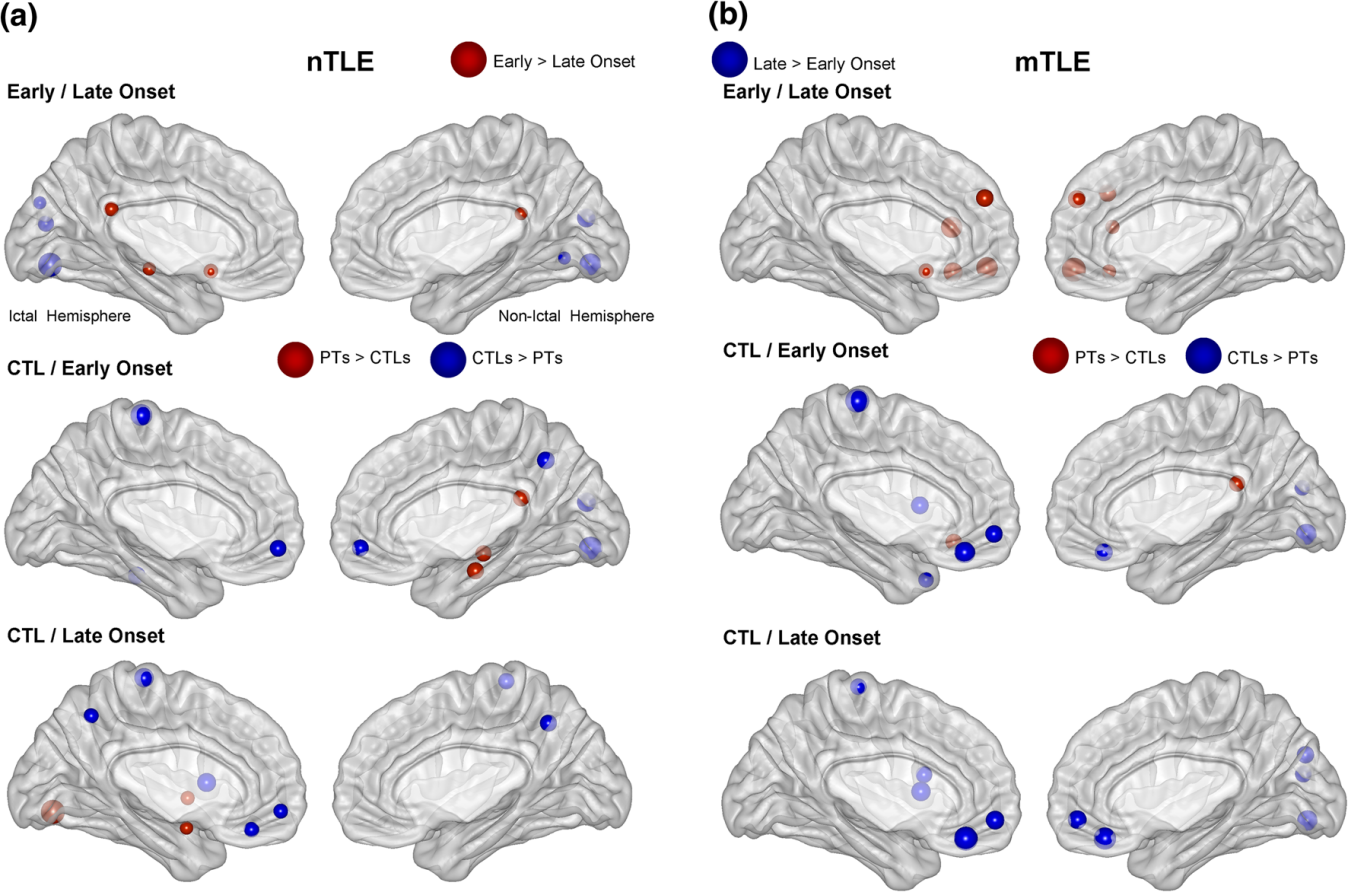
Description of the 10 largest clustering coefficient differences between the experimental groups, involving either the nTLE (**a**) or the mTLE (**b**) groups (Color figure online)

For each experimental group, we averaged the 5,000 CC values (or Eglob values, respectively) for each node (or edge, respectively). This resulted in one value for each node (or edge) and each group. Then, we computed the differences between the different groups’ values: EO-nTLE vs. LO-nTLE; EO-mTLE vs. LO-mTLE; EO-nTLE vs. NC; LO-nTLE vs. NC; EO-mTLE vs. NC; LO-mTLE vs. NC. For each comparison, the standard-deviation (SD) of the differences among the 116 regions was computed in order to show the regions with the most significant differences (mean ± 2 SD) (Fig. 1). Finally, for a more effective display, the ten largest differences between the groups involving the 90 cortical nodes were visualized with the BrainNet Viewer (http://www.nitrc.org/projects/bnv/) (Fig. 2, 3).

### Hippocampal Sub-Analysis

Lastly, because of the central role of the hippocampus in the underlying pathology of TLE, as well as to the ongoing process of epileptogenesis, we more closely examine the potentially unique networks effects of this structure. To accomplish this we focus on both the ictal and non-ictal hippocampus and compute the betweenness centrality (BC) of each with the rest of the brain (e.g. 115 regions), doing so within each group at each threshold. The BC is defined as the fraction of all shortest paths in the network that pass through a given node (Rubinov and Sporns 2010). In other words, this measure reflects how much a node is related to networks shortest paths, or how ‘central’ the node is to the main connections (Sequeira et al. 2013).

## Results

### Demographic Data

No significant difference were revealed between the patient groups for age, gender, and seizure laterality (*p* > 0.3). We found a significant difference for the illness (epilepsy) duration (*p* = 0.049) and age of onset variables (*p* = 0.035) between the mTLE and nTLE groups. The age of seizure onset was earlier in the mesial TLE than in the non-lesional TLE groups (Table 1).

### Whole-Brain Functional Analyses

At the whole-brain level, the bootstrapped analyses revealed significant differences between the experimental groups, for each property and for each threshold (Fig. 1; Supplementary Table 2 shows the F-values resulted from the one-way ANOVAs).

#### Modularity

Regarding the whole-brain modularity, we found that all the patient groups had a global reduced modularity, relative to the controls (Fig. 1a). In detail, while the late onset mesial TLE group (LO-mTLE) showed the largest reduction in modularity (less normative values), the early onset mesial TLE group (EO-mTLE) demonstrated the most normative values, closest to the the normal controls. The modularity differences between the early and late onset groups were larger within the mTLE, than within the nTLE group.

#### Clustering Coefficient

At the whole-brain level, the pattern of scores and the group differences for the CC measure were quite similar to those observed for the modularity (Fig. 1b; Sup. Table 2). In other words, the late onset mTLE group again showed the less normative (reduced) CC values relative to controls. The nTLE subgroups displayed the same profile that was evident for modularity, with the late onset nTLE group showing values closest to controls, and the early and late onset patients having relative close values. As was the case for modularity, in the setting of mTLE the two seizure onset groups demonstrated larger CC differences.

#### Global Efficiency

At the whole-brain level, differences between the groups on the Eglob measure were less obvious (Fig. 1c; Sup. Table 2), but still consistent with the previous two segregation measures. Indeed, we revealed similar effects for this integration measure: the late and early onset mTLE groups showed the largest differences, relative to the two nTLE subgroups, which showed closer values. The LO-mTLE group had the highest Eglob values for the sparsest matrices (thresholds 5–20 %) whereas the EO-nTLE group showed the highest values for the most connected matrices (thresholds 25–35 %), relative to the other groups. The controls demonstrated the lowest values for most of the thresholds tested, relative to the 4 TLE groups.

In summary, the differences between the experimental groups for both functional segregation and integration measures were concordant. We showed that, regardless of the presence of MTS, the 4 TLE groups had abnormal whole-brain functional integration (Eglob) and segregation (M and CC) values, through significant increase and reduction, respectively, relative to controls. The largest differences between early and late seizure onset groups were observed in the setting of mTLE. For most of the thresholds tested, the LO-mTLE had both the lowest segregation and the highest integration, whereas the EO-mTLE demonstrated more normative values.

### Local Results

**Fig. 4:**
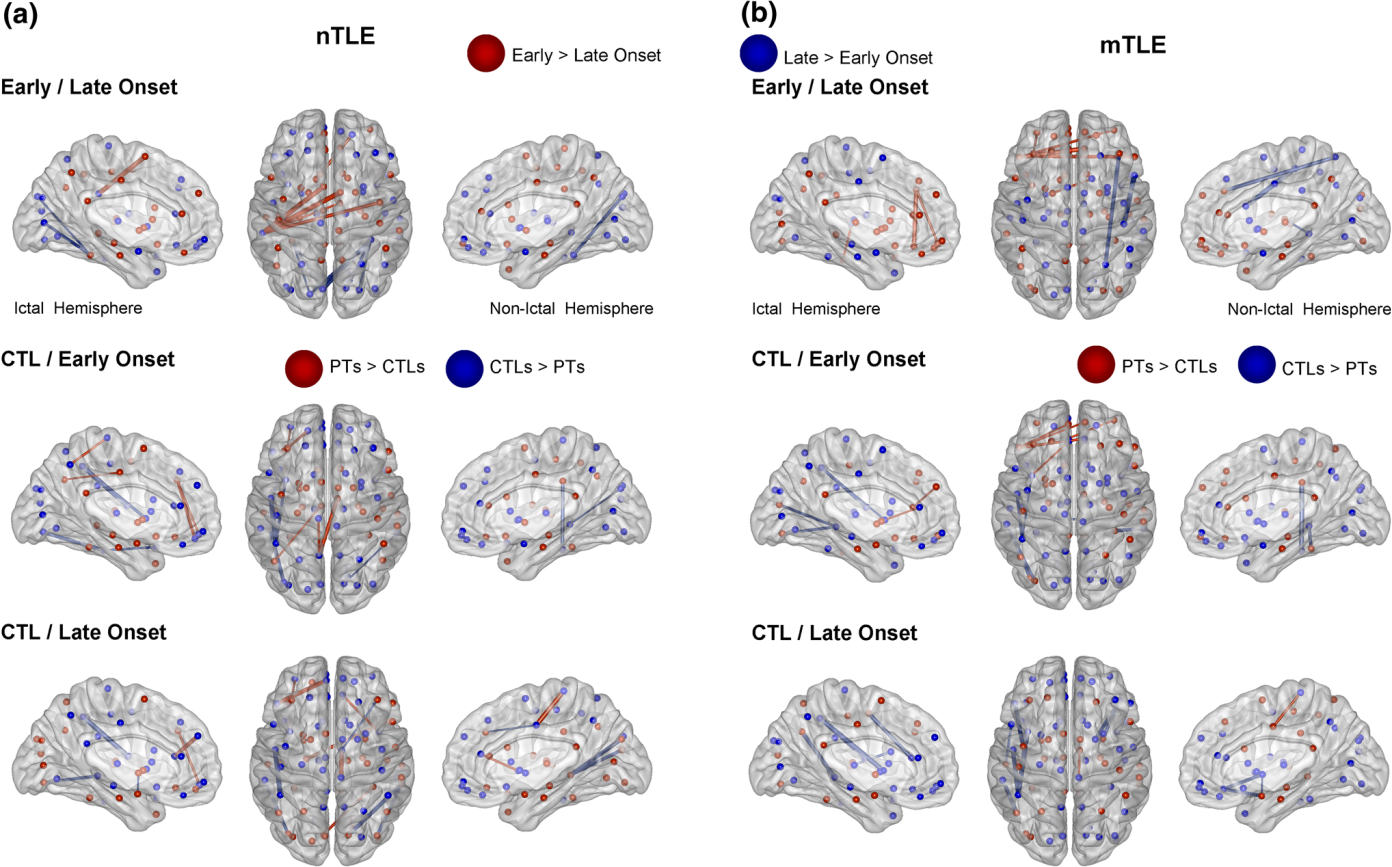
Description of the 10 largest global efficiency differences between the experimental groups, involving either the nTLE (**a**) or the mTLE (**b**) groups (Color figure online)

We investigated the regional changes between each patient group, relative to the control group. In other words, we explored which specific regions were affected in TLE, using the CC (Figs. 2, 3) and the Eglob (Fig. 4) values computed for each region and each link between pair of regions, respectively. For a better clarity, we will only describe the results obtained at the 15 % threshold, but the results for the other thresholds are displayed in Supplementary Fig. 1 and 2.

#### Clustering Coefficient

Figure 2 displays the CC differences for the 116 nodes, while Fig. 3 only shows the 10 largest differences between the cortical regions for the groups. Of note, the CC differences between the control and patient groups for the other thresholds are consistent with the results at the 15 % threshold (Supplementary Fig. 1).

### Non-Lesional TLE

#### Early Versus Late Onset

The comparison between EO and LO revealed that that the largest differences were bilaterally located in the occipital lobe with higher CC for the LO than the EO group (Figs. 2, 3). In contrast, the highest CCs for the EO group were located in medial regions such as the bilateral posterior cingulum (PCC), left olfactory cortex and parahippocampal gyrus, relative to the LO group.

#### Controls Versus Early Onset

The data revealed reduced CC in the frontal cortex for the EO nTLE group, relative to NC group. Also there were changes with reduced CC in the occipital lobe, paracentral lobule as well as the ictal temporal pole. In contrast, the EO group showed increased CC in 3 ROIs located in the contralateral/non ictal hemisphere: posterior cingulate cortex (PCC), paracentral gyrus and hippocampus (Fig. 3).

#### Controls Versus Late Onset

The strongest differences between the LO-nTLE and NC groups were mostly associated with reductions of CC in the ictal frontal cortex for the LO, relative to NC (Fig. 3). Also there were reductions of CC in the precuneus, caudate, and paracentral lobule for the LO group. In contrast, the LO group showed increased CC in 3 nodes in their ictal hemisphere: in the inferior occipital cortex, the pallidum and the amygdala.

### Mesial TLE

#### Early Versus Late Onset

When comparing the EO to the LO mTLE, the highest CC differences involved increased CC for the EO relative to the LO group. These differences were located bilaterally in the frontal cortex (Figs. 2, 3; Supplementary Fig. 1).

#### Controls Versus Early Onset

The comparison between the EO-mTLE and NC groups revealed that most of the strongest differences were associated with reduced CC in the frontal cortex for the EO relative to NCs, with this effect more prevalent in the ictal hemisphere (Fig. 3). Also, the EO group showed a CC reduction in the non-ictal occipital lobe, ictal paracentral lobule and temporal pole. In contrast, the patient group had one ROI with increased CC located in the contralateral PCC.

#### Controls Versus Late Onset

The LO group only demonstrated reduced CC in regions located in the frontal and occipital cortices, relative to controls.

In summary, with regard to the above local results involving CC, the 4 TLE patient groups showed common reductions of CC in the medial frontal regions as well as in the paracentral lobule, relative to controls (Fig. 2). In contrast, they also showed relative increases of CC in the MTL and especially in both parahippocampal gyri, the ictal amygdala, non-ictal hippocampus as well as the PCC, relative to controls.

### Global Efficiency

Figure 4 displays the 10 largest differences between cortical regions between the groups for the density threshold set at 15 %. However, all the Eglob differences between the groups are reported in the Supplementary Fig. 2.

### Non-lesional TLE

#### Early Versus Late Onset

When comparing the Eglob values between EO and LO-nTLE, the data revealed that the LO group had increased Eglob in the occipital lobe, relative to the EO group. In contrast, the EO group had the highest Eglob between inter-hemispheric regions, relative to the LO group.

#### Controls Versus Early Onset

The largest differences between the EO-nTLE and NC groups were located in the ictal hemisphere, involving multiple locations in every lobe. For instance, the patients showed increased Eglob in the ictal frontal cortex, and strong reduction of Eglob in more posterior regions, relative to the controls.

#### Controls Versus Late Onset

The differences of Eglob were also located in multiple regions, equally organized between both hemispheres. As for the EO-nTLE group, the LO patients had changes with increased Eglob in the frontal cortex and reduced Eglob in posterior regions. Indeed, occipital regions showed reduced Eglob with temporal regions in the patient relative to the control group.

### Mesial TLE

#### Early Versus Late Onset

The EO group demonstrated Eglob increases within the ictal frontal cortex and between both frontal cortices, relative to the LO group. In contrast, the LO group showed increased Eglob in the non-ictal hemisphere between the anterior and posterior regions, relative to the EO group.

#### Controls Versus Early Onset

For EO-mTLE, Eglob increases were present in the frontal cortex, within the ictal hemisphere, and also between both hemispheres, relative to controls. In contrast, reduced Eglob for the patients were evident mostly in posterior regions, involving the parietal, occipital and temporal lobes.

#### Controls Versus Late Onset

Most of the largest differences between the LO-mTLE and controls involved a reduction of Eglob for the patients. In detail, the LO group showed reduced Eglob between the frontal cortex and the temporal and parietal lobes. The largest reductions for the patients were located in the ictal hemisphere. Also, in contrast to the other patient groups, the LO-mTLE group did not demonstrate strong increased Eglob in the frontal cortices, relative to controls.

### Hippocampal Analysis

**Fig. 5:**
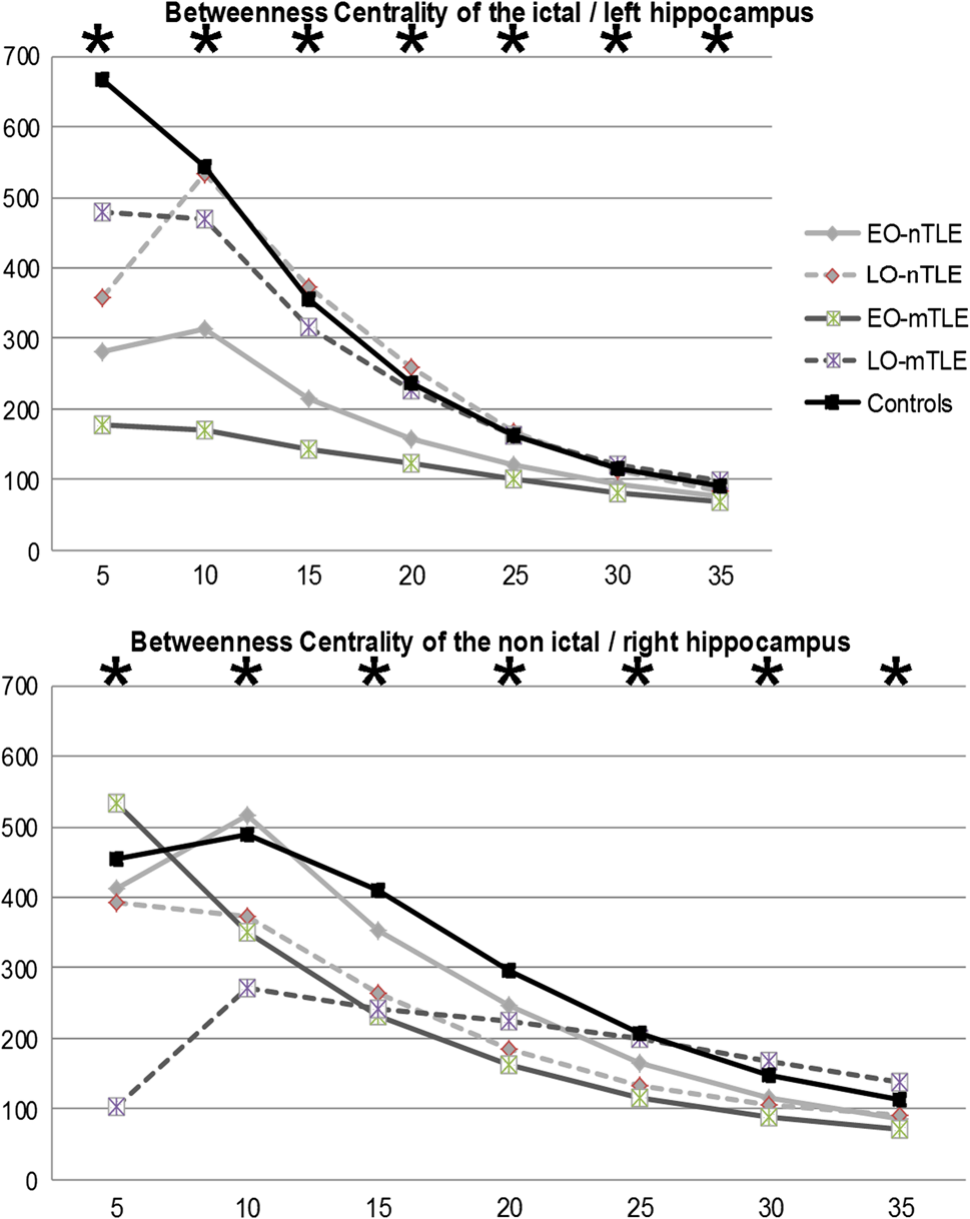
Betweenness centrality for each hippocampus. The graph theory metrics (y-axis) from the bootstrapped data are displayed across fixed density thresholds (x-axis) for binarized matrices. *Black line* control group, *Light gray lines* nTLE groups, *Dark gray lines* mTLE groups, *Bold lines* EO groups, *Dashed lines* LO groups. *Stars* indicate significant differences between groups; result issued from the ANOVAs realized between the 5 groups, for each threshold and hippocampus independently (*p* < 10^−12^)

Lastly, a specific analysis was conducted to investigate in our experimental groups connectivity differences emerging from a key structure at risk in TLE, namely the hippocampus. To accomplish this, we examined whether the centrality of the hippocampus, i.e., its strength as a network hub, varied as function of age of onset or the presence of lesional TLE. The measure, betweenness centrality (BC), was utilized (Fig. 5).

The results revealed that both EO groups showed the lowest BC for the ictal hippocampus, with the mTLE group displaying the weakest values, regardless of the statistical threshold. In contrast, both LO groups had higher and more normative BC values for their ictal hippocampus, though the normal controls showed higher values for most of the thresholds tested.

Regarding the non-ictal hippocampus, the EO-mTLE group had also the lowest BC values for every threshold, except the two more restrictive ones (5, 10 %), whereas the EO-nTLE group showed higher and more normative values for most of the thresholds tested. Of note, both EO groups had higher BC values for the non-ictal hippocampus than for the ictal hippocampus, a difference more prominent in the nTLE group. The LO-mTLE group showed more varied levels of BC at the different statistical thresholds for the non-ictal hippocampus, with low values at the more restrictive threshold (5, 10 %) and higher values for the less restrictive thresholds (30, 35 %). Finally, in contrast to the ictal hippocampus, the LO-nTLE showed reduced BC relative to both controls and EO-nTLE for the non-ictal hippocampus, for most of the thresholds.

## Discussion

The aim of this study was to investigate the resting-state FC organization in unilateral TLE patients, using graph theory parameters. We specifically explored the effect of early/late age of seizure onset, in the context of the presence of a temporal lesion or not. For that purpose, we analyzed parameters used in graph theory to define either brain functional segregation (M and CC) or integration (Eglob) at both the whole-brain and regional levels. Functional segregation has been defined as the “relative significant independence of small subsets in a system”, whereas functional integration represents “significant deviations from independence of large subsets” (Tononi et al. 1994). In other words, high segregation is related to a high independence between subsets of brain regions; while, in opposition, high integration means a high dependence between brain subsets. Previous studies have highlighted the utility of measuring several graph theory parameters in resting-state fMRI data to understand the complexity of the effect of TLE pathology on the brain resting state activity (Zhang et al. 2011).

While we provide indication that resting-state brain FC is impacted by age of seizure onset and the presence of MTS, we cannot exclude the possibility that these effects are caused by other rival factors such as seizure severity, factors that we have not been taken into consideration in the present study. This and other limitations will be discussed at the end this section. With these limitations in mind, it is clear that further resting-state fMRI investigations, are needed to understand and partition the complex and interacting influences of these factors in resting functional connectivity, particularly in the setting of TLE.

Overall, the present results show new evidence that TLE disrupts whole-brain and regional resting state FC, independently of the presence of MTS or the age of SO. Indeed, we observed that each TLE patient group was associated with both reduced functional segregation and increased integration in the entire brain, relative to normals. These results are consistent with previous studies such as Liao et al. (2010) that also demonstrated that both segregation was lower and integration was higher in mesial TLE patients than in healthy controls. It is important to note that their study was limited to TLE patients with MTS, thus, our findings broaden our understanding of diminished segregation in TLE to now include non-lesional patients. Overall, we demonstrate increased whole-brain integration among the network systems, an important finding that speaks to the possibility that forces of abnormal coherence and integration are at work in the epileptic brain, even in focal epilepsy such as TLE.

Our data also revealed three important differences between the experimental groups. One, we highlight small differences in whole-brain properties between early and late onset patients in the setting of non-lesional TLE, whereas in mesial TLE the early and late onset patients show large whole-brain differences, with the LO-mTLE having the less normative values. Previous studies have shown that mTLE patients more commonly have cognitive, structural and functional abnormalities than non-lesional TLE patients (e.g. Concha et al. 2009). Thus, our data are partially concordant with these results, as the LO-mTLE patients had less normative whole-brain organization than both nTLE groups, even though these three patient groups have similar illness duration and age. Two, we showed that when the TLE onset comes early in life, the presence of MTS may be mitigated, at least in terms of whole-brain organization. Such data is concordant with the notion that early onset epilepsies are associated with compensatory mechanisms as the younger brain is more plastic and can adapt more easily than an adult mature brain (Helmstaedter et al. 2004). This idea may also explain our results regarding the LO-mTLE patients and their less normative brain organization. Therefore, our data suggest that risk accrues for MTS patients whose TLE develops in adulthood, because the mature, injured brain may have lost its capacity to generate adaptive mechanisms to compensate for the deleterious effect of TLE on brain network organization. Another possibility is that the adult injured brain needs more time to adapt to the MTS pathology and, thus, the altered whole-brain organization in our later onset patients reflects their not being given enough time to develop compensatory processes. In this regard, it is important to note that illness duration for the LO-mTLE group is three times shorter than for the EO mTLE group. Three, the small differences revealed for the graph theory properties between EO and LO nTLE suggest that the age of seizure onset has little progressive impact on brain functional connectivity when there is no focal lesion present. However, ignoring lesion status, the LO group showed a slightly more normative segregation level than the EO group, with this effect independent of illness duration (which does not differ between the two groups). One explanation for this is that late onset seizures may not have impaired the non-lesional brain as much as early onset seizures. Noting that our data show a more harmful effect for LO seizures on brain organization in the mTLE group, our results indicate that there may be an interaction between age of onset and lesional status. This finding is consistent with the hypothesis sustained by Mueller et al. (2009), indicating that TLE with and without MTS are two distinct syndromes.

We also investigated regional network differences between our experimental groups, using clustering coefficient and global efficiency properties. The data highlight that the groups differ on both properties, not just in the ictal temporal lobe, but also other lobes, including the contralateral hemisphere. These findings are concordant with previous studies investigated small-world properties in TLE patients (Liao et al. 2010; Zhang et al. 2011). In detail, we revealed that the majority of local, regional group differences are located in the frontal cortex, for both integration and segregation levels. Indeed, we highlight a general pattern of increased segregation and integration within the frontal cortex and reduced integration between the frontal cortex and the rest of the brain for the patients, relative to the controls. However, within this larger group pattern of abnormality relative to controls there were important patient group differences. For instance, EO-mTLE was the only group to show increased integration between the frontal lobes, compared to the controls. We suggest that such an increase may be a sign of adaptive compensation within the frontal cortex, relative to the rest of the brain. In contrast, the LO-mTLE group showed both abnormally reduced segregation of the frontal cortex and reduced integration of the frontal cortex with the rest of the brain (especially in the ictal hemisphere). Specifically, the LO-mTLE had lower CC in the frontal cortex, relative to both controls and EO-mTLE. This result may suggest that adult age onset emerging from lesional temporal epilepsy causes higher levels of network impairment. Also, our data are consistent with the previous study done by Liao et al. (2010), which demonstrated that mTLE patients show a significant reduction in FC between several frontal regions, relative to controls. The implication of such frontal lobe abnormalities are large, as extemporal cognitive abnormalities have been reported in unilateral, focal TLE (Stretton and Thompson 2012), but discovering neurobiological evidence that might explain these cognitive (i.e., executive function) abnormalities has been difficult.

In terms of regional effects, we also observed several important findings involving the regional network effects between the early and late SO, regardless of the presence of a temporal lesion. For instance, TLE patients with an early onset showed increased CC in the PCC, relative to controls, regardless of lesional status. This is consistent with previous findings (Zhang et al. 2010, 2011). These authors suggest that the PCC is involved in the initiation of spike and slow-wave discharge activity, leading to an up-regulated network in these patients (Zhang et al. 2011). An alternative possibility is that increased CC in this specific region serves an adaptive purpose in EO patients, as the PCC has been described as one of the major hubs in the brain for cognitive processing (Buckner et al. 2009), resting-state activity (Fransson and Marrelec 2008), as well as a crucial structure for maintaining an inhibitory surround in unilateral, focal TLE (Tracy et al. 2012). For both purposes, an up-regulation of PCC connectivity would prove adaptive. Our data also provide evidence of increased CC in all groups relative to controls emerging from the non-ictal temporal lobe, with this effect most prominent for early onset patients. Lastly, it should be noted our, as well as others, findings of an abnormal degree of connectivity involving the hippocampus and the PCC provides evidence supporting the claim that the well-known default-mode network is dysfunctional in TLE patients (Zhang et al. 2010).

Finally, based on these observations of abnormal segregation in the MTL, we specifically investigated changes in the centrality of each hippocampus with the rest of the brain, as a function of age of SO and MTS presence. We observed that both TLE patients with early onset showed reduced centrality, relative to the other groups, suggesting that even though the nTLE patients do not have structural abnormalities evident on MRI, their ictal hippocampus demonstrates functional abnormality. Thus, these results point to the possibility that early TLE onset impairs a key feature of network status, “hubness”, in the ictal hippocampus to a more severe degree than late TLE onset, regardless of the presence of MTS. Indeed, this negative impact seems time-limited as nTLE and mTLE patients with a late seizure onset did not show such a BC reduction. Previous studies have shown that the severity of MTS is positively correlated with the age of seizure onset (Davies et al. 1996). Our data suggest that the effect of MTS on network development impact also varies by onset age. At first glance, this result appears contradictory with the whole-brain results we describe earlier as the LO-mTLE patients had the less normative integration and segregation values. However, we see this difference as pointing out a probable interaction between pathology and age of onset in network development and plasticity. Late onset seizures may impair both local and whole brain properties in the context of MTS, suggesting that structural abnormalities limit brain plasticity when seizures appear late but not early in life. Thus, late onset seizures have a more limited affect on connections between the ictal hippocampus and the rest of the brain, suggesting that they are more preserved than in the case of early onset seizures. Indeed, the loss of centrality for the ictal hippocampus (again regardless of the presence of structural abnormality) indicates that the hippocampus is more isolated in terms of regional connections. We propose that our data may reflect a process whereby early seizures allow developmental processes of network organization, i.e., segregation into modules, to proceed normally in regions outside the ictal temporal lobe, however, the pathology of mesial structures impedes hippocampal connectivity. In contrast, with late onset seizures local mesial structures may reap the benefit of having had years of healthy functioning, and, therefore, show adequate regional connectivity to the hippocampus, but, surprisingly, whole brain organization and modularity suffers.

Some limitations need to be highlighted. First, we were not able to separate patients based on their pathology side as the sample size would have been too small for meaningful statistical analyses. However, we believe combining the right and left TLE patients into one group remains an effective method of revealing whole-brain changes associated with TLE pathology. As a matter of fact, previous studies applied the same strategy (Sequeira et al. 2013; Maccotta et al. 2013), demonstrating significant effects of TLE on brain regional activity, using single photon emission computed tomography or fMRI, respectively. As described in previous papers (Doucet et al. 2013; Maccotta et al. 2013), differences between right and left TLE do not always reach significance due to low power and small sample size. This would likely be the case in the present study as the sample size of both the RTLE and LTLE groups is lower than 10. Finally, it should be noted that the major purpose of this paper was to focus on the global effect of the TLE pathology on the brain, regardless of the epileptogenic side. We believe that unilateral TLE causes functional changes, changes that are not specific to the side of the pathology and epileptic focus. These are the changes we wanted to investigate in this study.

Our method provides a depiction of whole brain functional connectivity in TLE, and is the first to focus on potential differences between the early and late onset forms of the disorder, with additional examination of the mediating effect of MTS. Second, we were not able to match the EO and LO mTLE patient groups on their illness duration, or to apply the same age cutoff for TLE onset between nTLE and mTLE. Indeed, we were not able to recruit many nTLE patients with younger illness onset, which makes sense as lesional pathology tends to lead to clinical symptoms earlier. This observation is consistent with previous studies showing that patients with MTS have a younger age of onset than patients without MTS (Davies et al. 1996). These authors also found that younger age at seizure onset was significantly correlated with the presence of more severe hippocampal sclerosis, suggesting the less mature hippocampus is more susceptible to insults. Importantly, in the present study, among the measures used hippocampal BC would appear most directly reflective of hippocampal integrity. Here, while, we did observe greater loss in BC for the EO group, this loss was not dependent on the presence of lesional pathology. Nevertheless, we acknowledge that we did not have a direct measure of hippocampal atrophy or MTS severity. Lastly, we chose to split the groups to achieve an equal number of patients in each EO/LO subgroup. With only a five-year difference in the age cutoff used, we suspect it is unlikely that all the differences we report are caused by these different thresholds.

Finally, it should be noted that it is possible that our patient groups do not match in terms of their seizure frequency or the total number of seizures in their lifetime. However, it is well known that patient ratings of seizure frequency and number are highly subjective and unreliable, as patients are often unaware of and have poor recall for their seizures, particularly when the inquiry is made many years later. Therefore, we chose to not take into account, nor include in our analyses, these measures.

## Conclusion

Our results confirm disturbed whole-brain FC in TLE, with diminished segregation and increased integration processes relative to controls. We provide evidence that the impact of age of SO on whole-brain resting-state FC depends on the presence of MTS. While we did our best to match the patient groups, we did not take into consideration other possible confounding factors such as seizure severity, a variable that is very difficult to quantify reliably. Nevertheless, we believe that the present work will open up a new window of investigation to further confirm or reject our interpretations about the effects of age of SO on resting-state brain activity.

We suggest that the presence of TLE seizures in the setting of a non-lesional brain has a less deleterious impact on whole-brain network organization than a lesional brain. Our data support the hypothesis that patients with early seizures onset are more prone to potential adaptive functional reorganization in extra-temporal regions than patients with late SO, with this early group increasing segregation in the non-ictal (contralateral) hippocampus and PCC, in particular, perhaps to ensure access to major healthy networks remote from seizure activity. Our data show for the first time that a late age of SO and the presence of MTS interact in important ways to alter most resting-state whole-brain functional properties. In contrast, at a local level, we revealed that the connectivity of the ictal hippocampus remains the most impaired for an early TLE onset, speaking to the network development “costs” of seizures, even in the absence of a temporal lobe lesion. By comparison, late onset TLE allows for some preservation of this mesial connectivity. Overall, our results highlight the importance of investigating the effect of seizure onset age when examining resting-state activity in TLE, as this factor appears to lead to different perturbations of network modularity and connectivity at the global and local level, with different implications for network plasticity and the preservation of adaptive organization.

## Electronic supplementary material

Below is the link to the electronic supplementary material.

Supplementary material 1 (PDF 759 kb)
