# Supplementary material for: Early and Late Age of Seizure Onset have a Differential Impact on Brain Resting-State Organization in Temporal Lobe Epilepsy
Source: Brain Topogr. 2014 Jun 1;28(1):113–26. doi: 10.1007/s10548-014-0366-6 (PMC4291512; doi:10.1007/s10548-014-0366-6)

G. Doucet, A. Sharan, D. Pustina, C. Skidmore, M. Sperling, J. Tracy “Early and late age of seizure onset have a differential impact on brain resting-state organization in temporal lobe epilepsy” *Brain Topography*.

Supplementary Table 1: Description of the 116 regions of interest (ROIs).

	**Abbreviation**	**Region**
1	FAG	Precentral_L
2	FAD	Precentral_R
3	F1G	Frontal_Sup_L
4	F1D	Frontal_Sup_R
5	F1OG	Frontal_Sup_Orb_L
6	F1OD	Frontal_Sup_Orb_R
7	F2G	Frontal_Mid_L
8	F2D	Frontal_Mid_R
9	F2OG	Frontal_Mid_Orb_L
10	F2OD	Frontal_Mid_Orb_R
11	F3OPG	Frontal_Inf_Oper_L
12	F3OPD	Frontal_Inf_Oper_R
13	F3TG	Frontal_Inf_Tri_L
14	F3TD	Frontal_Inf_Tri_R
15	F3OG	Frontal_Inf_Orb_L
16	F3OD	Frontal_Inf_Orb_R
17	ORG	Rolandic_Oper_L
18	ORD	Rolandic_Oper_R
19	SMAG	Supp_Motor_Area_L
20	SMAD	Supp_Motor_Area_R
21	COBG	Olfactory_L
22	COBD	Olfactory_R
23	FMG	Frontal_Sup_Medial_L
24	FMD	Frontal_Sup_Medial_R
25	FMOG	Frontal_Med_Orb_L
26	FMOD	Frontal_Med_Orb_R

27	GRG	Rectus_L
28	GRD	Rectus_R
29	ING	Insula_L
30	IND	Insula_R
31	CIAG	Cingulum_Ant_L
32	CIAD	Cingulum_Ant_R
33	CINMG	Cingulum_Mid_L
34	CINMD	Cingulum_Mid_R
35	CIPG	Cingulum_Post_L
36	CIPD	Cingulum_Post_R
37	HIPPOG	Hippocampus_L
38	HIPPOD	Hippocampus_R
39	PARA_HIPPOG	ParaHippocampal_L
40	PARA_HIPPOD	ParaHippocampal_R
41	AMYGDG	Amygdala_L
42	AMYGDD	Amygdala_R
43	V1G	Calcarine_L
44	V1D	Calcarine_R
45	QG	Cuneus_L
46	QD	Cuneus_R
47	LINGG	Lingual_L
48	LINGD	Lingual_R
49	O1G	Occipital_Sup_L
50	O1D	Occipital_Sup_R
51	O2G	Occipital_Mid_L

52	O2D	Occipital_Mid_R
53	O3G	Occipital_Inf_L
54	O3D	Occipital_Inf_R
55	FUSIG	Fusiform_L
56	FUSID	Fusiform_R
57	PAG	Postcentral_L
58	PAD	Postcentral_R
59	P1G	Parietal_Sup_L
60	P1D	Parietal_Sup_R
61	P2G	Parietal_Inf_L
62	P2D	Parietal_Inf_R
63	GSMG	SupraMarginal_L
64	GSMD	SupraMarginal_R
65	GAG	Angular_L
66	GAD	Angular_R
67	PQG	Precuneus_L
68	PQD	Precuneus_R
69	LPCG	Paracentral_Lobule_L
70	LPCD	Paracentral_Lobule_R
71	NCG	Caudate_L
72	NCD	Caudate_R
73	NLG	Putamen_L
74	NLD	Putamen_R
75	PALLG	Pallidum_L
76	PALLD	Pallidum_R

77	THAG	Thalamus_L
78	THAD	Thalamus_R
79	HESCHLG	Heschl_L
80	HESCHLD	Heschl_R
81	T1G	Temporal_Sup_L
82	T1D	Temporal_Sup_R
83	T1AG	Temporal_Pole_Sup_L
84	T1AD	Temporal_Pole_Sup_R
85	T2G	Temporal_Mid_L
86	T2D	Temporal_Mid_R
87	T2AG	Temporal_Pole_Mid_L
88	T2AD	Temporal_Pole_Mid_R
89	T3G	Temporal_Inf_L
90	T3D	Temporal_Inf_R
91	CERCRU1G	Cerebelum_Crus1_L
92	CERCRU1D	Cerebelum_Crus1_R
93	CERCRU2G	Cerebelum_Crus2_L
94	CERCRU2D	Cerebelum_Crus2_R
95	CER3G	Cerebelum_3_L
96	CER3D	Cerebelum_3_R
97	CER4_5G	Cerebelum_4_5_L
98	CER4_5D	Cerebelum_4_5_R
99	CER6G	Cerebelum_6_L
100	CER6D	Cerebelum_6_R
101	CER7BG	Cerebelum_7b_L

102	CER7BD	Cerebelum_7b_R
103	CER8G	Cerebelum_8_L
104	CER8D	Cerebelum_8_R
105	CER9G	Cerebelum_9_L
106	CER9D	Cerebelum_9_R
107	CER10G	Cerebelum_10_L
108	CER10D	Cerebelum_10_R
109	VER1_2	Vermis_1_2
110	VER3	Vermis_3
111	VER4_5	Vermis_4_5
112	VER6	Vermis_6
113	VER7	Vermis_7
114	VER8	Vermis_8
115	VER9	Vermis_9
116	VER10	Vermis_10

Abbreviations: Inf=Inferior; Mid=Middle; Sup=Superior; Oper=Opercular; Orb=Orbital; Tri=Triangularis; L=Left; R=Right.

Supplementary Table 2: Averaged whole-brain values for each property, each threshold and each group, resulted from the bootstrapped analyses. The F-value results from the one-way ANOVA realized for each threshold.

Density	EO-nTLE	LO-nTLE	EO-mTLE	LO-mTLE	CTL	F-value
	**Modularity**					
**5%**	0.6719	0.6917	0.669	**0.6603**	0.6813	1097
**10%**	0.5544	0.5658	0.5682	**0.5398**	0.5761	6044
**15%**	0.483	0.4873	0.4972	**0.4585**	0.5086	18052
**20%**	0.418	0.4256	0.4349	**0.398**	0.4503	22376
**25%**	0.3623	0.3717	0.379	**0.3456**	0.4009	27586
**30%**	0.3203	0.324	0.329	**0.3032**	0.3523	27891
**35%**	0.2847	0.2825	0.285	**0.2673**	0.3077	23499
	**Clustering Coefficient**					
**5%**	0.3945	0.424	0.3947	**0.3857**	0.4132	2487
**10%**	0.4598	0.4606	0.4454	**0.4397**	0.4752	7269
**15%**	0.4743	0.4761	0.4643	**0.4489**	0.4859	14711
**20%**	0.4833	0.4894	0.4813	**0.4624**	0.4991	20289
**25%**	0.4925	0.5021	0.4963	**0.4789**	0.5134	24829
**30%**	0.5042	0.5154	0.5114	**0.4976**	0.5287	29685
**35%**	0.5198	0.5306	0.528	**0.5183**	0.5451	33152
	**Global Efficiency**					
**5%**	0.2709	0.262	0.2696	**0.2743**	0.2637	1399
**10%**	0.4225	0.4242	0.4221	**0.4262**	0.4218	1121
**15%**	0.503	0.5048	0.501	**0.5083**	0.5012	8836
**20%**	0.5622	0.5613	0.5595	**0.5639**	0.5586	7316
**25%**	**0.6074**	0.6066	0.6048	0.6069	0.6029	12057
**30%**	**0.6411**	0.6408	0.6399	0.6403	0.639	9573
**35%**	**0.6682**	0.6681	0.6679	0.668	0.6677	2495

Bold values indicate the TLE group that differ the most from the control group, for each threshold.

G. Doucet, A. Sharan, D. Pustina, C. Skidmore, M. Sperling, J. Tracy “Early and late age of seizure onset have a differential impact on brain resting-state organization in temporal lobe epilepsy” *Brain Topography*.

Supplementary Figure 1: Clustering Coefficient Differences between the experimental groups, for the 116 ROIs, for each density threshold (except 15%, which is displayed in Figure 2B).

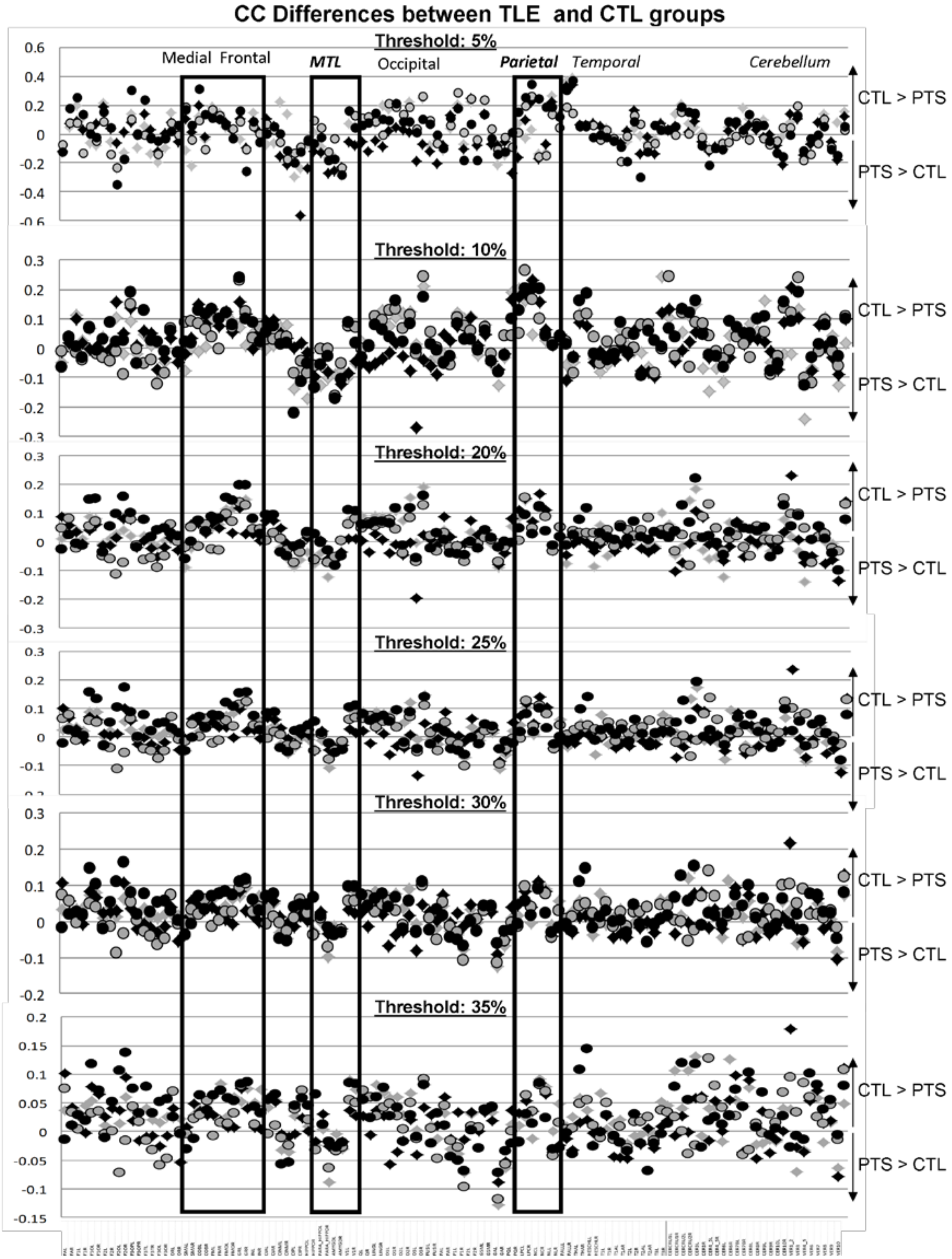

G. Doucet, A. Sharan, D. Pustina, C. Skidmore, M. Sperling, J. Tracy "Early and late age of seizure onset have a differential impact on brain resting-state organization in temporal lobe epilepsy" *Brain Topography*.

Supplementary Figure 2: Global Efficiency Differences between the experimental groups, for the 116 ROIs, for each density threshold.

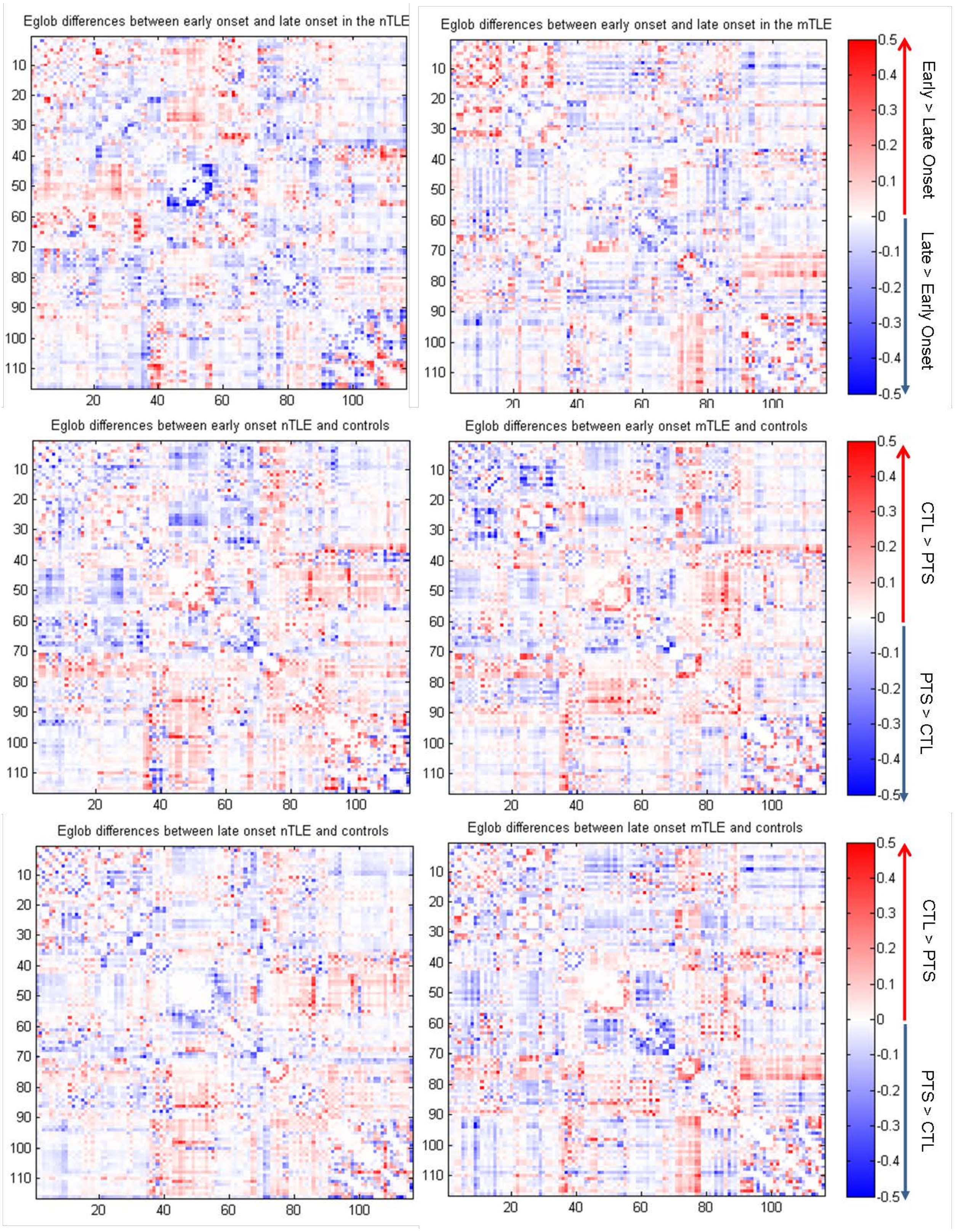

Supplement: Supplementary file 1 — Supplementary material 1 (PDF 759 kb) [file 10548_2014_366_MOESM1_ESM.pdf]
